# Gene- and immune-targeted therapy combinations using dual-matched biomarkers for patient selection

**DOI:** 10.1038/s41698-025-01038-w

**Published:** 2025-07-24

**Authors:** Daisuke Nishizaki, Razelle Kurzrock, Jacob J. Adashek, Ki Hwan Kim, Hyo Jeong Lim, Mina Nikanjam, Ramez N. Eskander, Paul T. Fanta, Ryosuke Okamura, Suzanna Lee, Jason K. Sicklick, Scott M. Lippman, Shumei Kato

**Affiliations:** 1 Center for Personalized Cancer Therapy and Division of Hematology and Oncology, Department of Medicine, University of California San Diego, Moores Cancer Center, La Jolla, CA USA; 2 MCW Cancer Center and Genomic Sciences and Precision Medicine Center, Medical College of Wisconsin, Milwaukee, WI USA; 3 WIN consortium, Paris, France; 4 Department of Oncology, The Sidney Kimmel Comprehensive Cancer Center, The Johns Hopkins Hospital, Baltimore, MD USA; 5 Division of Hematology and Medical Oncology, Department of Internal Medicine, Seoul National University Boramae Medical Center, Seoul, Republic of Korea; 6 Department of Internal Medicine, Veterans Health Service Medical Center, Seoul, Republic of Korea; 7 Center for Personalized Cancer Therapy and Division of Gynecologic Oncology, Department of Obstetrics, Gynecology, and Reproductive Sciences, University of California San Diego, Moores Cancer Center, La Jolla, CA USA; 8 Department of Surgery, Kyoto University Hospital, Kyoto, Japan; 9 Division of Surgical Oncology, Department of Surgery, Center for Personalized Cancer Therapy, University of California San Diego, La Jolla, CA USA

**Keywords:** Cancer genomics, Tumour biomarkers, Biomarkers, Cancer

## Abstract

Combinations of gene-targeted therapy and immune checkpoint inhibitors (ICIs) have been conducted, though generally without biomarker-based patient selection for both therapy types. We evaluated outcomes of 17 patients with advanced cancers treated with both targeted agents and ICIs, matched to distinct genomic and immune biomarkers, from a cohort of 715 cases discussed at our Molecular Tumor Board. Despite 29% of patients having undergone ≥3 prior therapies, the disease control rate (includes SD ≥ 6 months or objective response) was 53%, with a median progression-free survival (PFS) of 6.1 months (95% CI, 2.9–not estimable) and median overall survival (OS) of 9.7 months (95% CI, 6.7–not estimable). Three patients (~18%) achieved prolonged PFS and OS (PFS: 23.4+, 33.0, 59.7 months; OS: 23.4+, 43.6, 62.1+ months) in B-cell lymphoma unclassifiable, ovarian, and gastroesophageal cancers. Median dosages were 100% for ICIs and 50% for gene-targeted agents, with Grade 3–4 serious adverse events occurring in 24%. We additionally conducted a database search to evaluate the prevalence of biomarker-based dual therapy trials, which revealed only 1.3% (4/314) of such clinical trials included biomarkers for both targeted therapies and ICIs. These findings highlight the potential of dual biomarker-matched combination therapy even after multiple therapy lines and support further investigation of dual-matched therapy.

## Introduction

Over the past two decades, the deployment of gene- and immune-targeted therapies has revolutionized the therapeutic landscape for both solid and hematologic malignancies. Historically, using one gene-targeted agent to impact one molecular alteration led to meaningful clinical benefit for patients, but this strategy has its limitations, as resistance usually develops. Even so, the exploitation of next-generation sequencing (NGS) to identify potential actionable biomarkers has informed novel treatment strategies for patients with cancers and led to a multitude of gene-targeted approvals by The Food and Drug Administration (FDA). NGS has also identified immunotherapeutic biomarkers, including tumor mutational burden (TMB), mismatch repair deficiency (dMMR), and microsatellite instability (MSI)^1,2^, which can be targeted with immune checkpoint inhibitors (ICIs).

Strategies using ICIs have generally been used as single agent and usually in later lines of therapy. Over the last several years, efforts to include immunotherapy in the treatment-naïve setting as well as in combination with gene-targeted therapies has offered higher response rates as well as higher complete remission rates^3^. However, despite the improvements in response rates and survival outcomes, many of these studies do not include a biomarker for inclusion^4^ and a majority of patients do not benefit.

Evidence that certain genomic alterations lead to increased expression of various immunoregulatory molecules is of interest for combining gene- and immune-targeted therapeutics^5^. Moreover, in cancers such as melanoma, FDA approval of atezolizumab (programmed cell death ligand 1 [PD-L1] inhibitor), cobimetinib (MEK inhibitor), and vemurafenib (*BRAF* V600E inhibitor) for advanced *BRAF* V600-mutated melanoma illustrates the value of combining these modalities^6^. This approach is an example of using a genomic biomarker for inclusion (*BRAF* V600E); however, there was no specific inclusion criteria for patients to receive atezolizumab. Indeed, rarely do trials include a biomarker for immunotherapy and it is even less common to perform trials to include a biomarker for both a targeted therapy and an immunotherapy when both drugs are combined^4^. This is especially important since not all patients derive clinical benefit and the potential risk for side effects from immunotherapies is both significant and able to occur at any time point after receiving therapy^7^.

We sought to identify patients who were presented at The University of California, San Diego (UCSD) Molecular Tumor Board (MTB) and were given immunotherapy combined with gene-targeted therapies based on their cancers’ immune and genomic markers. Herein, clinical outcomes including responsiveness, survival, and toxicities from dual-matched therapies were assessed.

## Results

### Patient characteristics

Among 429 patients who were assessable for therapeutic clinical outcome after MTB discussion^8^, 17 patients were eligible for the present analysis. Patient flow diagram is provided in Supplementary Fig. 1. The median age was 67 years (range 20–86), and 9 (53%) of them were female. Approximately 30% of the patients (29% [5/17]) received three or more lines of therapy before undergoing dual-matched therapy. Treatments were administered between October 2016 and August 2018. The most common cancer diagnosis was gastroesophageal cancer (24% [4/17]) and hematologic malignancies (24% [4/17]), followed by bladder/ureter cancer (18% [3/17]) and gynecologic cancer (18% [3/17]). Upon treatment recommendation by MTB, 5 of 17 patients (29%) received all recommended agents, and the other (71% [12/17]) received part of MTB recommended agents (Supplementary Table 1). All patients with solid tumors had advanced metastatic disease (i.e., not oligometastatic), and all patients with lymphoma showed diffuse organ involvement.

### Each patient had a biomarker-based rationale for the utilization of both gene-targeted agents and ICIs

**Fig. 1:**
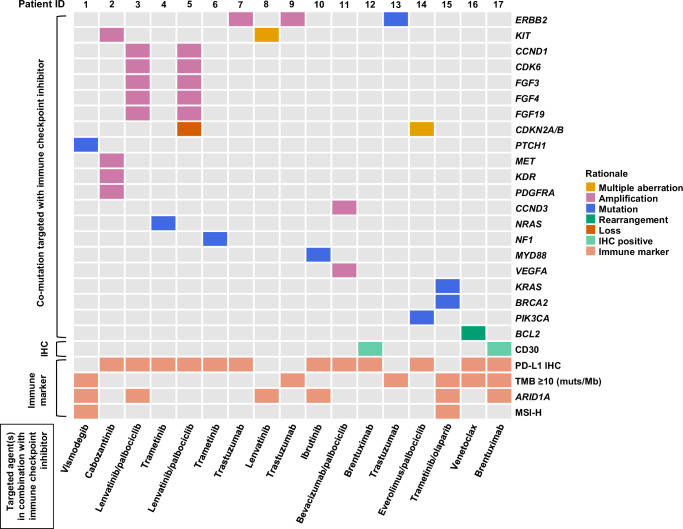
The rationale for the dual-matched therapy with ICI and targeted agent. This heatmap illustrates gene aberrations and the corresponding targeted agents utilized for treatment. Each column within the heatmap represents an individual patient. For instance, patient #1 was treated with vismodegib for *PTCH1* mutation. Additionally, patient #1 exhibited positive immune markers, including TMB ≥ 10 (muts/Mb), an *ARID1A* alteration, and MSI-high, and thus, received immunotherapy. ICI immune checkpoint inhibitor, IHC immunohistochemistry, Mb megabase, MSI microsatellite instability, muts mutations, TMB tumor mutation burden.

**Fig. 2:**
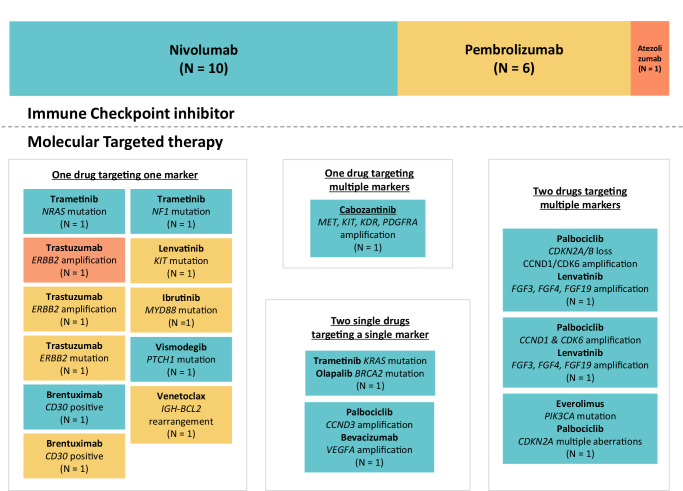
Utilization of genomically targeted drugs and immune checkpoint inhibitors in the context of dual-matched therapy. The upper segment of the figure depicts the types of the immune checkpoint inhibitors implemented in the dual-matched therapy. Nivolumab and pembrolizumab are anti-PD-1 agents; atezolizumab is an anti-PD-L1 agent. The lower portion of the figure depicts individualized boxes representing the targeted agent(s) used in dual-matched therapy. The background colors of molecular targeted agent(s) denote the simultaneously administered immune checkpoint inhibitor with the same background color (blue, nivolumab; yellow, pembrolizumab; orange, atezolizumab). This tailored combination approach was customized for each patient’s unique and complex molecular profile. Patients could receive a single drug that targeted more than one biomarker (since small molecule inhibitors often have multiple targets) or more than one drug that targeted the same biomarker.

The biologic rationale for the selection of administered agents is outlined in Table 1^9,10^ and depicted in Fig. 1, based on molecular profiling by NGS and IHC. Each patient had at least one predictive immune biomarker for ICIs (Fig. 1): Eight patients had only PD-L1 IHC as an immune biomarker; four had positive PD-L1 IHC along with other immune markers; and five had only markers other than PD-L1 IHC that were positive (e.g., high TMB or MSI-high or *ARID1A* alterations). As to genomic aberrations, the patterns of aberrations were mostly distinct from patient to patient. Only two out of 17 patients had the same genomic aberration patterns (*ERBB2* amplification), although their co-positive immune markers were different. Nivolumab (anti-PD-1) was the most frequently used ICI (59% [10/17]). Figure 2 illustrates the targeted agents utilized for treatment and their combination patterns with ICIs. Detailed information regarding genomic aberrations, overexpression, TMB, MSI status, and PD-L1 IHC is provided in Supplementary Table 2.

**Table 1:** Characteristics of 17 patients who underwent dual matched targeted agent and immune checkpoint inhibitor simultaneously

ID (age/sex)	Cancer type	ECOG-PS	Number of lines of therapy	Dual-matched treatment	Rationale for ICI	Rationale for targeted therapy	Best response	PFS (Mos)	OS (Mos)	Comment
1 (67/F)	Gynecologic (endometrial cancer)	0	4	Nivolumab Vismodegib	Nivolumab for TMB 12 Muts/Mb, MSI-high, *ARID1A* G95fs*10	Vismodegib (hedgehog inhibitor) for *PTCH1* S1203fs*52	SD	8.3	37.7+	TMB 12 (Muts/Mb), MSI-high
2 (40/M)	Glioblastoma	3	1	Nivolumab Cabozantinib	Nivolumab for PD-L1 positive (IC 5% by SP142) and *PD-L1/L2* amplification	Cabozantinib (multi-kinase inhibitor including for MET, KIT, KDR and PDGFRA) for *MET, KIT, KDR* and *PDGFRA* amplifications	SD	6.1	15.1	TMB 2 (Muts/Mb)
3 (60/M)	Gastro-esophageal	1	2	Nivolumab Lenvatinib Palbociclib	Nivolumab for PD-L1 positive (TC 10% IC 1% by SP142), *ARID1A* R1276*	Palbociclib (CDK4/6 inhibitor) for *CCND1* and *CDK6* amplifications Lenvatinib (multikinase inhibitor including for FGFR axis) for *FGF3, FGF4*, and *FGF19* amplifications	PD	0.9	2.6+	TMB 8 (Muts/Mb)
4 (84/F)	Peripheral T cell lymphoma	0	2	Trametinib Nivolumab	Nivolumab for PD-L1 positive (TC 1% by SP142)	Trametinib (*MEK* inhibitor) for *NRAS* Q61R	SD	6.1	6.7	TMB 6 (Muts/Mb)
5 (54/M)	Gastro-esophageal	1	1	Nivolumab Lenvatinib Palbociclib	Nivolumab for PD-L1 positive (Tumor cell 1%, immune cell 1% by SP142 antibody)	Palbociclib (CDK4/6 inhibitor) for *CDKN2A/B* loss and *CCND1* and *CDK6* amplifications (all of which elevate CKD4.6 Lenvatinib for *FGF3, FGF4*, and *FGF19* amplifications	PD	2.6	2.6	TMB 7 (Muts/Mb)
6 (68/F)	Gynecologic (high grade serous adenocarcinoma of ovary)	0	1	Trametinib Nivolumab	Nivolumab for PD-L1 positive (IC 5% by SP142)	Trametinib (MEK inhibitor) for *NF1* R440*	PR	33.0	43.6	TMB and MSI not available
7 (86/M)	Bladder/Ureter	1	2	Atezolizumab Trastuzumab	Atezolizumab for PD-L1 positive (IC 5% by SP142)	Trastuzumab (HER2 inhibitor) for *ERBB2* amplification	PD	2.9	5.1+	TMB 7 (Muts/Mb)
8 (72/F)	Gastrointestinal stromal tumor	1	4	Pembrolizumab Lenvatinib	Pembrolizumab for *ARID1A* truncation exon 18	Lenvatinib (multi-kinase inhibitor including for KIT) for *KIT* K558_E562del, N822K, V654A	SD	12.9+	12.9+	TMB 7 (Muts/Mb)
9 (80/M)	Bladder/Ureter	0	2	Pembrolizumab Trastuzumab	Pembrolizumab for TMB 18 muts/Mb	Trastuzumab (HER2/ERBB2 antibody) for *ERBB2* amplification	PD	4.7	9.7	TMB 18 (Muts/Mb)
10 (51/F)	Gastro-esophageal	1	4	Pembrolizumab Ibrutinib	Pembrolizumab for *ARID1A* Q1519fs*13 and PD-L1 positive (CPS ≥ 50 by 22C3)	Ibrutinib for *MYD88* S219C^9,10^	PR	59.7	62.1+	TMB and MSI not available
11 (67/M)	Gastro-esophageal	1	1	Nivolumab Bevacizumab Palbociclib	Nivolumab for PD-L1 positive (TC 5% by SP142)	Palbociclib (CDK4/6 inhibitor) for *CCND3* amplification Bevacizumab (VEGF antibody) for *VEGFA* amplification	CR	7.7	8.0	TMB 5 (Muts/Mb)
12 (20/F)	Cutaneous T cell lymphoma	2	5	Brentuximab Nivolumab	Nivolumab for PD-L1 positive (TC > 25%, IC > 25% by SP142)	Brentuximab (CD30 antibody) for CD30 positive by IHC	PD	2.2	2.2	TMB 7 (Muts/Mb)
13 (69/F)	Bladder/Ureter	1	4	Pembrolizumab Trastuzumab	Pembrolizumab for TMB 14 muts/Mb	Trastuzumab (HER2/ERBB2 antibody) for *ERBB2* S310F	SD	6.3	15.7	TMB 14 (Muts/Mb)
14 (61/F)	Gynecologic (vulvar cancer)	1	2	Nivolumab Palbociclib Everolimus	Nivolumab for PD-L1 positive (IC 5% by SP142)	Everolimus (mTor inhibitor) for *PIK3CA* E545K Palbociclib (CDK4/6 inhibitor) for multiple alterations in *CDKN2A (*which elevate CDK4/6)	PD	2.1	2.1	TMB 8 (Muts/Mb)
15 (69/F)	Malignant mixed Müllerian tumor	1	3	Nivolumab Trametinib Olaparib	Nivolumab for MSI-high, TMB 36 Muts/Mb, *ARID1A* P2005fs*10	Trametinib (MEK inhibitor) for *KRAS* G12A Olaparib (PARP inhibitor) for *BRCA2* T3033fs*29	PD	3.6	3.7	TMB 36 (Muts/Mb), MSI-high
16 (72/M)	Diffuse large B cell lymphoma	2	2	Pembrolizumab Venetoclax	Pembrolizumab for PD-L1 positive (TPS 60% by 22C3), TMB 14 Muts/Mb	Venetoclax (BCL-2 inhibitor) for *IGH-BCL2* rearrangement	PD	1.0	2.4	TMB 14 (Muts/Mb)
17 (37/M)	B cell lymphoma, unclassifiable	0	2	Brentuximab Pembrolizumab	Pembrolizumab for PD-L1 positive (TC 50%, IC 10% by SP142), TMB 16 Muts/Mb, *ARID1A* G149fs*77	Brentuximab (CD30 antibody) for CD30 positive by IHC	PR	23.4+	23.4+	TMB 16 (Muts/Mb)

*CR* complete response, *ECOG-PS* Eastern Cooperative Oncology Group Performance Status, *IC* immune cell, *ICI* immune checkpoint inhibitor, *Mos* month, *MSI* microsatellite instability, *Muts/Mb* mutations per megabase, *NE* not evaluable, *PD* progressive disease, *PR* partial response, *SD* stable disease, *TC* tumor cell, *TMB* tumor mutation burden.

### Response to dual-matched therapy

**Fig. 3:**
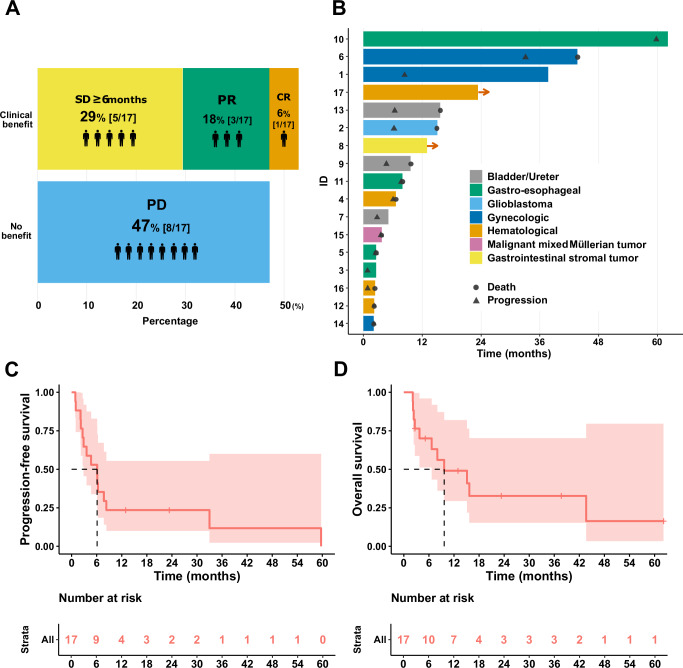
Clinical outcomes of the dual-matched therapy. **A** Clinical benefit rate (SD ≥ 6 months/CR/PR) of the 17 patients who underwent dual-matched therapy was 53% (9/17). **B** The swimmer’s plot provides information on the duration of survival and the timing of progression for each patient. The arrow indicates that the patients were progression-free at the date of their last follow-up. Two patients did not show apparent progression, but their therapy was suspended due to an adverse event (ID 8) or lost to follow-up (ID 17). **C**, **D** Kaplan–Meier curves for progression-free survival (PFS, Panel **C**) and overall survival (OS, Panel **D**) of 17 patients who received dual-matched therapy for treatment-refractory diverse cancers. Median follow-up period was 8.1 months (range 2.1–62.1). The pale red area indicates the 95% confidence interval (CI). Median PFS was 6.1 months (95% CI, 2.9–not estimable), and median OS was 9.7 months (95% CI, 6.7–not estimable). CNS central nervous system, CR complete response, PD progressive disease, PR partial response, SD stable disease.

Clinical benefit (defined as stable disease (SD) ≥ 6 months as well as complete or partial response (CR or PR)) were seen in nine of 17 patients (53%): one, with complete response (6% [1/17]); three, had partial response (18% [3/17]), five, stable disease ≥6 months (29% [5/17]). Eight patients had progressive disease (47% [8/17]). Overall, clinical benefit was observed in 53% [9/17] of the patients (Fig. 3A).

### Survival analysis

Figure 3B displays patients’ time to progression and last follow-up. Median follow-up period was 8.0 months (range 2.2–62). Three patients (ID 6, 10, 17) had a durable PFS (more than 18.3 months [three times the median PFS]) **(**PFS of 33.0, 59.7 and 23.4+ months) and four patients (ID 1, 6, 10, and 17) had a prolonged survival (more than 19.3 months [two times the median OS]) (OS of 37.7+, 43.6, 62.1+ and 23.4+ months). Three patients with durable response in both PFS and OS (ID 6, 10, 17) survived more than 3 years after the initiation of dual-matched therapy. Kaplan–Meier curves showed that median PFS was 6.1 months (95% confidence interval [CI], 2.9–not estimable [NE]; Fig. 3C) and median OS was 9.7 months (95% CI, 6.7–NE; Fig. 3D).

### Dosing of the dual-matched therapy and adverse events

**Fig. 4:**
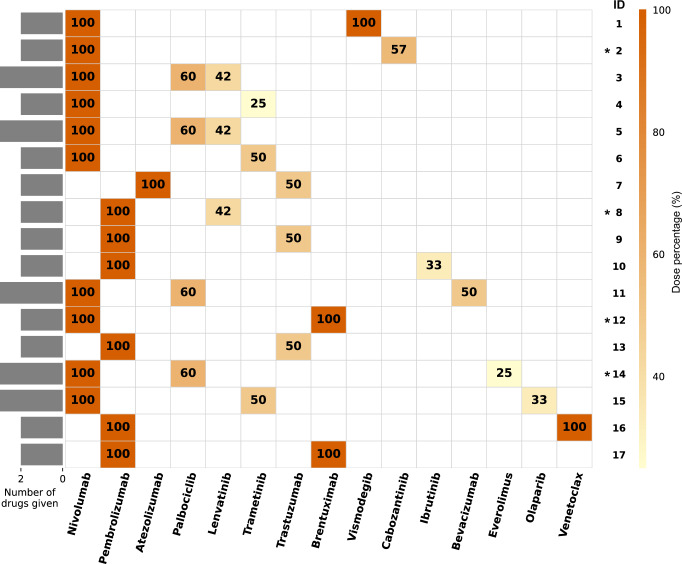
Dose percentage of each administered agent. This heatmap describes the initial dose percentage of each drug given. Each cell contains dose percentage with its corresponding color. Each row indicates each patient, and each column indicates each administered agent. Dose percentage of immune checkpoint inhibitors (*N* = 17) was 100% in all patients, and that of targeted agents (*N* = 22) was 50% (median) and ranged from 25% to 100%. Patients whose ID are with an asterisk had serious adverse events (Grade ≥3) during their treatment course.

We assessed the actual dose of each administered agent as a percentage of the FDA-approved dose. Dose percentage of ICI (*N* = 17) administered was 100% in all patients, and that of gene-targeted agents was 50% (median) (range, 25–100%) (Supplementary Table 3; and Fig. 4); physicians routinely started with a lower dose of the gene-targeted agent to avoid toxicity for the combination. Serious adverse events (SAEs), at least possibly related to the dual-matched therapy, occurred in four patients (24% [4/17]): one had Grade 4 (encephalitis and sepsis), while the other three had Grade 3 (acute kidney injury [*N* = 1], hypothyroidism [*N* = 1], pancytopenia [*N* = 1]). All four were hospitalized, but none of the cases were fatal. The causes of hospitalization included acute kidney injury, which necessitated a 75% dose reduction of cabozantinib (ID 2); hypothyroidism combined with poor compliance in taking levothyroxine, resulting in the termination of pembrolizumab (ID 8); sepsis and encephalitis, leading to the discontinuation of the therapy (ID 12); and pancytopenia, which led to the suspension of palbociclib with recovery of blood counts (ID 14). During their course of therapy, six patients (35% [6/17]) had to reduce their treatment doses.

### Trial search identified only a small portion of trials that employed biomarkers for both gene-targeted therapy and immunotherapy in patient selection

**Fig. 5:**
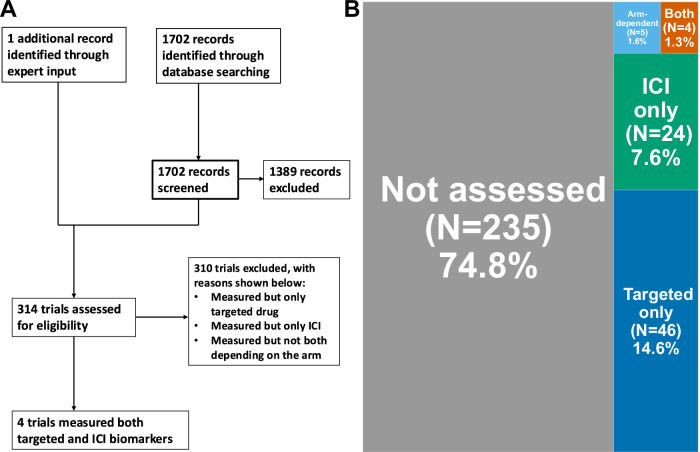
Trial search results. **A** PRISMA flow chart of the trial search (See Methods). Search was conducted on Mar 24, 2023, for ClinicalTrials.gov, using the search terms “(PD-1 OR PD-L1 OR CTLA4 OR LAG3) AND (targeted OR combination)” with a start date of Jan 1, 2018. After the screening, 1389 records did not meet the inclusion criteria and were therefore excluded from the further assessment. **B** Proportion of trials that utilized biomarkers for both the immune checkpoint inhibitor and the gene-targeting agent. Of the 314-trials assessed, 235 trials (75% [235/314]) did not assess biomarkers for patient inclusion. Forty-six trials checked gene alterations or immunohistochemistry for the targeted gene. The details of the four trials that assessed biomarkers for both the immune- and the gene-targeted therapy are presented in Supplementary Table 4. ICI immune checkpoint inhibitor.

Our initial search (conducted March 24, 2023; ClinicalTrials.gov) yielded a total of 1702 registered clinical trials. Based on screening and one additional record identified through expert input, 314 trials appeared to be eligible for further evaluation (see Methods) and were assessed in detail (Fig. 5A). Among these 314 trials included for detailed assessment, only 1.3% (4/314) of trials employed a biomarker for patient inclusion for both the ICI and the gene-targeted agent (Fig. 5B). Targeted genes were *HER2* in two trials, *KRAS* G12C in one trial, and *BRAF* V600E in one trial. All four trials assessed immune markers: three assessed the PD-L1 positivity for patient inclusion (one trial requires PD-L1 TPS ≥ 1%, and two trials with any PD-L1 positivity) while one required MSI-high/dMMR. Meanwhile, 75% (235/314) of the trials did not assess any biomarkers for patient inclusion. The details of four included trials are shown in Supplementary Table 4.

## Discussion

The present study demonstrated the clinical outcomes and safety of combined genomic and immune-matched therapy—dual-matched therapy—in a pan-cancer setting. Both targeted agents and ICIs were selected with a specific biomarker-based rationale in each patient. Despite multiple lines of previous therapy (29% [5/17] had ≥3 lines of therapy), the disease control rate (includes SD ≥ 6 months or objective response) was 53%. The 17 patients analyzed in the present study represents only a fraction (2.4%) of the total patients (*N* = 715) presented during the study period at MTB meetings^8^; however, our findings indicated that a certain proportion of patients may derive benefits from dual-matched therapy.

In the present analysis, we focused on patients who received biomarker-based targeted therapy and immunotherapy––the dual-matched therapy––upon MTB recommendations. The MTB took into account the fact that advanced cancers are individually distinct and complex when it comes to genomic and immune markers^11^, and facilitates the best treatment options with in-depth assessment of actionable biomarkers and other patient-specific information^12^. In particular, our prior studies show that targeting tumors with a higher degree of drug matching to alterations (reflected by a higher Matching Score) was associated with better clinical outcomes^13,14^. Overall, eight of 17 patients showed clinical benefit (53%); one patient attained a complete response; three, a partial response; and five had stable disease for 6 months or more. This is despite the fact that half of them (4/8) having had ≥3 lines of prior therapy. Targeted genes included *CCND3, MYD88, NF1, KIT, ERBB2, NRAS*, and *PTCH1*. Survival outcomes were comparable to those reported in observational studies evaluating second-line or later immunotherapy^15,16^. Among the eight patients with clinical benefit, four patients had durable survival exceeding 1.5 years.

Genomic alterations in metastatic cancers are diverse, distinct, and unique—akin to malignant snowflakes^11^. The present study is no exception; all 17 patients had unique genomic aberration patterns, which were matched in an N-of-1 approach (Fig. 1). Specific rationales to select treatment drugs are of the essence, and every drug given had a biomarker-based rationale (Fig. 2). In addition to genomic alterations, immunotherapy biomarkers were fully considered. In this analysis, PD-L1 IHC was positive in 71% (12/17) of patients^17,18^; TMB ≥ 10 (muts/MB), in 35% (6/17)^19,20^; *ARID1A* mutation, in 35% (6/17)^21^; and MSI-high in 12% (2/17)^2,22^. Of note, six patients had multiple positive immune markers; this highlights the importance of incorporating comprehensive immune analysis. Appreciation of the unique footprint of each cancer provides clues to effectively combat the disease itself. Molecular profiling can shed light on the precise evaluation of these footprints, and our data showcases an example of exploiting these clues across cancer types.

FDA-approved immune markers currently used to predict the response to anti-PD-1/PD-L1 therapy include PD-L1 IHC, TMB, and MSI status. Although a high PD-L1 IHC score generally predicts a positive response, as shown in this analysis, some patients with low PD-L1 expression still respond to immunotherapy^17^. In contrast, a high PD-L1 expression score does not guarantee a response to anti-PD-1 therapy, perhaps because of intratumoral heterogeneity. In the present study, 8 of the 17 patients were positive for PD-L1 IHC alone, without other immune markers; of these, 4—including one with a high PD-L1 IHC score (>25% TC)—did not attain clinical benefit. PD-L1 is used as one of the biomarkers for immunotherapy, but it has limitations^17^. Regarding MSI, only about 2% of all tumors are MSI-high^23^. High TMB is predictive of therapeutic efficacy, yet there is ongoing debate regarding the threshold for its clinical utility^1,24,25^. In the present study, the clinical decision to use immunotherapy was mainly based on such available markers. Indeed, these biomarkers have led to selection of patients who may potentially benefit from immunotherapy, as certain patients experienced a durable response. However, it is known that each tumor may have a unique portfolio of immune markers^26^. To further personalize cancer immunotherapy, it is essential to explore beyond existing biomarkers, including transcriptomic analysis using RNA sequencing^27–29^ and analysis of soluble immune markers^30^ to tailor immunotherapy treatments. With the expanding use of agents targeting CTLA-4, LAG-3, and TIGIT, a key question is whether biomarker-based selection will improve outcomes. As predictive markers remain limited for these newer targets, further data are needed to guide precision immunotherapy.

One patient with MSI-high and high TMB (36 mutations/Mb) did not respond to PD-1 blockade (ID 15). This patient harbored a truncating *JAK1* mutation, potentially impairing interferon-γ signaling and contributing to resistance^31^ despite strong indicators of immunogenicity. This case highlights the importance of identifying genomic alterations that may confer resistance to ICIs, even in tumors with otherwise favorable biomarkers. Systemic aggregation and evaluation of such discordant cases may help refine patient selection.

While simultaneous combination therapy for patients with dual-positive biomarkers may appear rational, it remains unclear whether it consistently yields superior outcomes compared to sequential approaches. Given the relatively higher rates of ≥G3 toxicity observed with the dual-matched therapies (24%) and the possibility of reduced dosing of targeted agents (Fig. 4), sequential treatment strategies might allow for full-dose administration, possibly with fewer adverse events and without compromising effectiveness. Although historically it was combination therapies that led to cures in childhood lymphomas and leukemias^32^, further studies will need to address the benefits of combined versus sequential therapy.

Our clinical trial search revealed that only a small portion (~1.3%) of the 314 clinical trials found in ClinicalTrials.gov used biomarkers both for targeted agents and for immunotherapy in patient enrollment. Since the introduction of ICIs, the idea of augmenting the effectiveness of gene-targeted therapies with ICIs has naturally arisen, usually based on preclinical data. Various trials have been conducted, yet there has been little implementation of combined gene-targeted therapies based on clear biomarker-based evidence along with ICIs selected based on immunomic markers, as our systematic search showed. The lessons learned from our prior MTBs, and suggested herein, are that biomarker-based combination treatment may correlate with better outcomes than standard monotherapies or unmatched therapies typically based on non-biomarker-driven trials^8^.

Further studies with a basket design or platform design may be helpful in combining targeted and immunotherapy markers. Basket studies targeting mutations such as *BRAF* V600E, *RET*, *NTRK*, and immune markers such as high TMB or MSI-high have been reported. However, most studies are designed to impact one target at a time^25,33^. A platform designed trial could allow for patients to receive a targeted therapy up front based on a biomarker and if no response or progression and the tumor has an immune biomarker adding an immunotherapy to the regimen. Other such studies could try to compare patients with both immune and targeted therapy markers who receive a customized combination versus investigators choice of targeted therapy, immunotherapy, or broad-spectrum chemotherapy (e.g., carboplatin/paclitaxel, gemcitabine/cisplatin). A study such as the latter could help elucidate if there is a survival advantage to individualizing therapy for patients based on their genomic profiles. However, because drug availability for individual patients may vary and be limited, and because the biomarker field is moving forward quickly, a randomized trial may be difficult to operationalize.

One of the real-world limitations for MTB-directed therapy includes drug availability and its acquisition process. Limitations of the current study include lack of a control group, small sample size, retrospective nature of the analysis, and tumor heterogeneity. Given the period during which patients were enrolled in this study, potential biomarkers for ICI response such as interferon-γ-related signatures^34^ or melanoma-based nomograms^35^, as well as certain immune evasion-related gene alterations (e.g., *B2M* alteration), could not be incorporated. Despite the limitations, the strength of our study lies in the fact that molecular profiling was exploited to choose drugs. While our study was based on tumor-only panel data, we acknowledge that matched tumor-normal sequencing may enhance the interpretation of genomic data^36^, and that the integration of RNA sequencing may play an increasingly important role in future precision oncology assays^12,36^. Also, while our current framework excluded chemotherapy-based regimens and concomitant radiotherapy due to the lack of predictive biomarkers, future studies will need to evaluate their roles when used alongside targeted therapies. In particular, the emergence of antibody–drug conjugates emphasizes the need to re-evaluate the role of the chemotherapy backbone within the evolving framework of precision medicine^37^.

In summary, our data demonstrate the potential effectiveness of dual-matched therapy, using a biomarker-based approach for combining gene-targeted agents and ICIs. Even so, there has been limited use of biomarkers for patient inclusion in recently conducted clinical trials, per our clinical trials search. Further prospective trials should be initiated in order to further elucidate how biomarker-based treatment selection can enable optimization of individualized combination cancer therapy.

## Methods

### Patient selection

From December 2012 to September 2018, 715 therapy-evaluable patients with diverse treatment-refractory cancers were presented at Molecular Tumor Boards at UC San Diego Moores Cancer Center^8^. Among these, patients who received ICIs combined with gene-targeted agents based on the following rationale were included in this analysis: (i) harboring TMB ≥ 10 Muts/Mb, MSI-high, positive PD-L1 immunohistochemistry (IHC), or an *ARID1A* gene alteration^21^; and (ii) harboring gene alteration(s) or protein overexpression(s) corresponding to the targeted agents used for (e.g. HER2 positive (*ERBB2* mutations/amplifications or immunohistochemistry 3+) being targeted by anti-HER2 therapy). The expression level of PD-L1 with IHC was evaluated by the SP142 assay (Ventana Medical Systems, Inc., Tucson, Arizona, USA) or the 22C3 assay (Dako North America, Inc., Carpinteria, California, USA). PD-L1 status was decided based on the proportion of tumor area occupied by PD-L1-expressing tumor-infiltrating immune cell (% IC) or the percentage of PD-L1 expressing tumor cells (% TC) when evaluated with the Ventana PD-L1 SP142 assay, and was deemed positive if either percentage was ≥1%. For the 22C3 assay, tumor proportion score (TPS) or combination proportion score (CPS) was used, which we deemed positive if either score was ≥1%. All NGSs and IHCs were performed at Clinical Laboratory Improvement Amendments (CLIA)-certified laboratories. All investigations were conducted in accordance with the guidelines of the Institutional Review Board of University of California, San Diego for data collection (Study of Personalized Cancer Therapy to Determine Response and Toxicity, UCSD_PREDICT, NCT02478931) and for any investigational interventions for which patients or their legal guardians provided written informed consent. The study protocol was approved by the Institutional Review Board of University of California San Diego (UCSD Project 130794), and informed consent was obtained from all participants involved in the study. All procedures were carried out in accordance with the principles of the Declaration of Helsinki.

### Definition of matched therapy

In this study, “matched” targeted therapy using a tyrosine kinase inhibitor (TKI) or other small molecule inhibitor and/or a monoclonal antibody (mAb) was defined as administration based on target gene alterations by NGS or overexpression detected by NGS or IHC. Matched immunotherapy with ICI was defined as administration of ICI for positive PD-L1 by IHC, TMB ≥ 10 (muts/MB), MSI-high, or *ARID1A* aberrations^21^. “Dual-matched” combination of targeted therapy and immunotherapy was defined here as simultaneous administration of both agents with a biomarker for both the targeted therapy and the ICI.

### Dosing information and assessment of safety

We defined dose percentage as the proportion of actual dose given compared to the FDA-approved dose. If a single drug has several approved doses amongst cancer types, we used the highest approved dose as a denominator. Adverse event severity was assessed using the Common Terminology Criteria for Adverse Events (CTCAE) v4.0^38^.

### Search for clinical trials that investigate “dual-matched” approach targeting both genomic and immune markers

We conducted a database search to identify ongoing or completed clinical trials investigating a combination therapy of immunotherapy and targeted therapy for patients with neoplasms. We searched US National Institutes of Health Ongoing Trials Register (ClinicalTrials.gov) during the period from 1 Jan 2018 to 24 Mar 2023, employing the search terms “ (PD-1 OR PD-L1 OR CTLA4 OR LAG3) AND (targeted OR combination).” The yielded records of registered clinical trials were screened by the authors, using Rayyan^39^. Clinical trials investigating the simultaneous combination of targeted agents and ICIs were included. The exclusion criteria were: (i) trials incorporating concomitant radiotherapy or systemic cytotoxic chemotherapy in their treatment regimens, even if immunotherapy and targeted therapy were administered simultaneously; (ii) trials administering an investigational immunomodulatory drug, even if they assessed a combination with currently available ICIs and targeted drug(s); and (iii) trials without assessment of both genomic/protein and immune analysis for patient enrollment.

### Outcome endpoints and statistical analysis

All patients were evaluated for the treatment response in accordance with RECIST 1.1 criteria. Progression-free survival (PFS) was defined as the interval between the date of treatment initiation and the earliest occurrence of disease progression or death from any cause. Overall survival (OS) was calculated as the duration between the date of treatment commencement and the last recorded follow-up or death. Data cutoff date was June 30, 2023. Patients still progression-free (for PFS) or alive (for OS) at time of last contact or data cut-off date, whichever was earlier, were censored at that time point. Durable response was defined as a patient with a PFS that exceeded three times the median PFS of all patients; durable or prolonged OS was defined as survival that exceeded two times the median OS of all patients in this cohort (per the definition of Pons-Tostivint et al.)^40^. A two-sided *p* value < 0.05 was deemed statistical significance. All statistical analyses were performed using R 4.2.3 (R Foundation for Statistical Computing, Vienna, Austria) and Python 3.10.5.

## Supplementary information

Supplemental Tables and Figures

## Data Availability

Raw data for this study were generated at UC San Diego. Derived data supporting the findings of this study are available from the corresponding author upon request.

## Code availability

The underlying code for this study is not publicly available but may be made available to qualified researchers on reasonable request from the corresponding author.

## References

[1] Goodman, A. M. et al. Tumor mutational burden as an independent predictor of response to immunotherapy in diverse cancers. *Mol. Cancer Ther.* **16**, 2598–2608 (2017). 28835386 10.1158/1535-7163.MCT-17-0386 PMC5670009

[2] Le, D. T. et al. PD-1 blockade in tumors with mismatch-repair deficiency. *N. Engl. J. Med.* **372**, 2509–2520 (2015). 26028255 10.1056/NEJMoa1500596 PMC4481136

[3] Motzer, R. et al. Lenvatinib plus pembrolizumab or everolimus for advanced renal cell carcinoma. *N. Engl. J. Med.* **384**, 1289–1300 (2021). 33616314 10.1056/NEJMoa2035716

[4] Adashek, J. J., Goloubev, A., Kato, S. & Kurzrock, R. Missing the target in cancer therapy. *Nat. Cancer* **2**, 369–371 (2021). 34368781 10.1038/s43018-021-00204-w PMC8336921

[5] Szeto, C. W. et al. Association of differential expression of immunoregulatory molecules and presence of targetable mutations may inform rational design of clinical trials. *ESMO Open* **7**, 100396 (2022). 35158206 10.1016/j.esmoop.2022.100396 PMC8850727

[6] U.S. Food & Drug Administration. *FDA approves atezolizumab for BRAF V600 unresectable or metastatic melanoma*, https://www.fda.gov/drugs/resources-information-approved-drugs/fda-approves-atezolizumab-braf-v600-unresectable-or-metastatic-melanoma (2020).

[7] Zhou, S., Khanal, S. & Zhang, H. Risk of immune-related adverse events associated with ipilimumab-plus-nivolumab and nivolumab therapy in cancer patients. *Therapeutics Clin. Risk Manag.* **15**, 211–221 (2019). 10.2147/TCRM.S193338 PMC6362938 30774357

[8] Kato, S. et al. Real-world data from a molecular tumor board demonstrates improved outcomes with a precision N-of-One strategy. *Nat. Commun.* **11**, 4965 (2020). 33009371 10.1038/s41467-020-18613-3 PMC7532150

[9] Treon, S. P., Xu, L. & Hunter, Z. MYD88 mutations and response to Ibrutinib in Waldenström’s Macroglobulinemia. *N. Engl. J. Med.* **373**, 584–586 (2015). 26244327 10.1056/NEJMc1506192

[10] Ngo, V. N. et al. Oncogenically active MYD88 mutations in human lymphoma. *Nature* **470**, 115–119 (2011). 21179087 10.1038/nature09671 PMC5024568

[11] Kurzrock, R. & Giles, F. J. Precision oncology for patients with advanced cancer: the challenges of malignant snowflakes. *Cell Cycle* **14**, 2219–2221 (2015). 26030337 10.1080/15384101.2015.1041695 PMC4615125

[12] Rodon, J. et al. Genomic and transcriptomic profiling expands precision cancer medicine: the WINTHER trial. *Nat. Med.* **25**, 751–758 (2019). 31011205 10.1038/s41591-019-0424-4 PMC6599610

[13] Sicklick, J. K. et al. Molecular profiling of cancer patients enables personalized combination therapy: the I-PREDICT study. *Nat. Med.* **25**, 744–750 (2019). 31011206 10.1038/s41591-019-0407-5 PMC6553618

[14] Sicklick, J. K. et al. Molecular profiling of advanced malignancies guides first-line N-of-1 treatments in the I-PREDICT treatment-naïve study. *Genome Med.* **13**, 155 (2021). 34607609 10.1186/s13073-021-00969-w PMC8491393

[15] Ruiz-Patiño, A. et al. Immunotherapy at any line of treatment improves survival in patients with advanced metastatic non-small cell lung cancer (NSCLC) compared with chemotherapy (Quijote-CLICaP). *Thorac. Cancer* **11**, 353–361 (2020). 31828967 10.1111/1759-7714.13272 PMC6996989

[16] Punchhi, G., Hussein, A. & Kulkarni, S. Real-world survival outcomes of immunotherapy for advanced non-small cell lung cancer: A single-center retrospective review. *Thorac. Cancer* **15**, 394–401 (2024). 38239043 10.1111/1759-7714.15205 PMC10864119

[17] Patel, S. P. & Kurzrock, R. PD-L1 expression as a predictive biomarker in cancer immunotherapy. *Mol. Cancer Ther.* **14**, 847–856 (2015). 25695955 10.1158/1535-7163.MCT-14-0983

[18] Reck, M. et al. Pembrolizumab versus chemotherapy for PD-L1-positive non-small-cell lung cancer. *N. Engl. J. Med.* **375**, 1823–1833 (2016). 27718847 10.1056/NEJMoa1606774

[19] Marcus, L. et al. FDA approval summary: pembrolizumab for the treatment of tumor mutational burden-high solid tumors. *Clin. Cancer Res.* **27**, 4685–4689 (2021). 34083238 10.1158/1078-0432.CCR-21-0327 PMC8416776

[20] Marabelle, A. et al. Association of tumour mutational burden with outcomes in patients with advanced solid tumours treated with pembrolizumab: prospective biomarker analysis of the multicohort, open-label, phase 2 KEYNOTE-158 study. *Lancet Oncol.* **21**, 1353–1365 (2020). 32919526 10.1016/S1470-2045(20)30445-9

[21] Okamura, R. et al. ARID1A alterations function as a biomarker for longer progression-free survival after anti-PD-1/PD-L1 immunotherapy. *J. Immunotherapy Cancer* **8**, 10.1136/jitc-2019-000438 (2020). 10.1136/jitc-2019-000438 PMC7057434 32111729

[22] Marcus, L., Lemery, S. J., Keegan, P. & Pazdur, R. FDA approval summary: pembrolizumab for the treatment of microsatellite instability-high solid tumors. *Clin. Cancer Res.* **25**, 3753–3758 (2019). 30787022 10.1158/1078-0432.CCR-18-4070

[23] Latham, A. et al. Microsatellite instability is associated with the presence of lynch syndrome pan-cancer. *J. Clin. Oncol.* **37**, 286–295 (2019). 30376427 10.1200/JCO.18.00283 PMC6553803

[24] Prasad, V. & Addeo, A. The FDA approval of pembrolizumab for patients with TMB >10 mut/Mb: was it a wise decision? No. *Ann. Oncol.* **31**, 1112–1114 (2020). 32771305 10.1016/j.annonc.2020.07.001

[25] Friedman, C. F. et al. Atezolizumab treatment of tumors with high tumor mutational burden from MyPathway, a multicenter, open-label, phase IIa multiple basket study. *Cancer Discov.* **12**, 654–669 (2022). 34876409 10.1158/2159-8290.CD-21-0450 PMC9394388

[26] Kato, S. et al. Expression of TIM3/VISTA checkpoints and the CD68 macrophage-associated marker correlates with anti-PD1/PDL1 resistance: implications of immunogram heterogeneity. *Oncoimmunology* **9**, 1708065 (2020). 32117584 10.1080/2162402X.2019.1708065 PMC7028323

[27] Nesline, M. K. et al. PD-L1 Expression by RNA-sequencing in non-small cell lung cancer: concordance with immunohistochemistry and associations with pembrolizumab treatment outcomes. *Cancers* **15**, 10.3390/cancers15194789 (2023). 10.3390/cancers15194789 PMC10571724 37835483

[28] Adashek, J. J. et al. LAG-3 transcriptomic expression patterns across malignancies: Implications for precision immunotherapeutics. *Cancer Med.*10.1002/cam4.6000 (2023). 37132280 10.1002/cam4.6000 PMC10315766

[29] Nishizaki, D. et al. Viewing the immune checkpoint VISTA: landscape and outcomes across cancers. *ESMO Open* **9**, 10.1016/j.esmoop.2024.102942 (2024). 10.1016/j.esmoop.2024.102942 PMC10966162 38503143

[30] Hayashi, H. et al. Soluble immune checkpoint factors reflect exhaustion of antitumor immunity and response to PD-1 blockade. *J. Clin Invest.* **134**, 10.1172/jci168318 (2024). 10.1172/JCI168318 PMC10977985 38557498

[31] Zaretsky, J. M. et al. Mutations associated with acquired resistance to PD-1 blockade in melanoma. *N. Engl. J. Med.* **375**, 819–829 (2016). 27433843 10.1056/NEJMoa1604958 PMC5007206

[32] Frei, E. et al. Studies of sequential and combination antimetabolite therapy in acute leukemia: 6-mercaptopurine and methotrexate. *Blood* **18**, 431–454 (1961).

[33] Tateo, V. et al. Agnostic approvals in oncology: getting the right drug to the right patient with the right genomics. *Pharmaceuticals* **16**, 10.3390/ph16040614 (2023). 10.3390/ph16040614 PMC10144220 37111371

[34] Ayers, M. et al. IFN-γ-related mRNA profile predicts clinical response to PD-1 blockade. *J. Clin. Investig.* **127**, 2930–2940 (2017). 28650338 10.1172/JCI91190 PMC5531419

[35] Chatziioannou, E. et al. Nomogram for predicting survival after first-line anti-PD-1-based immunotherapy in unresectable stage IV melanoma: a multicenter international study. *ESMO Open* **9**, 103661 (2024). 39096893 10.1016/j.esmoop.2024.103661 PMC11345525

[36] Beaubier, N. et al. Integrated genomic profiling expands clinical options for patients with cancer. *Nat. Biotechnol.* **37**, 1351–1360 (2019). 31570899 10.1038/s41587-019-0259-z

[37] Shitara, K. et al. An open-label, randomized, multicenter, phase 3 study of trastuzumab deruxtecan (T-DXd) + chemotherapy (chemo) ± pembrolizumab (pembro) versus chemo + trastuzumab ± pembro in first-line metastatic HER2+ gastric or gastroesophageal junction (GEJ) cancer: DESTINY-Gastric05. *J. Clin. Oncol.* **43**, TPS4207 (2025).

[38] National Cancer Institute Division of Cancer Treatment & Diagnosis. *Common Terminology Criteria for Adverse Events (CTCAE), Protocol Development, CTEP*, https://ctep.cancer.gov/protocoldevelopment/electronic_applications/ctc.htm (2021).

[39] Ouzzani, M., Hammady, H., Fedorowicz, Z. & Elmagarmid, A. Rayyan—a web and mobile app for systematic reviews. *Syst. Rev.* **5**, 210 (2016). 27919275 10.1186/s13643-016-0384-4 PMC5139140

[40] Pons-Tostivint, E. et al. Comparative analysis of durable responses on immune checkpoint inhibitors versus other systemic therapies: a pooled analysis of phase III trials. *JCO Precis. Oncol.* **3**, 1–10 (2019). 35100670 10.1200/PO.18.00114

