# Supplementary material for: Gene- and immune-targeted therapy combinations using dual-matched biomarkers for patient selection
Source: NPJ Precis Oncol. 2025 Jul 24;9:253. doi: 10.1038/s41698-025-01038-w (PMC12290096; doi:10.1038/s41698-025-01038-w)
Supplement: Supplementary file 1 — Supplemental Tables and Figures [file 41698_2025_1038_MOESM1_ESM.pdf]

SUPPLEMENT

Gene- and Immune-Targeted Therapy Combinations Using Dual-Matched Biomarkers for Patient Selection

Daisuke Nishizaki[1#], Razelle Kurzrock[2,3#], Jacob J. Adashek[4], Ki Hwan Kim[5], Hyo Jeong Lim[6], Mina Nikanjam[1], Ramez N. Eskander[7], Paul T. Fanta[1], Ryosuke Okamura[8], Suzanna Lee[1], Jason K. Sicklick[9], Scott M. Lippman[1], and Shumei Kato[1]

# Equally contributed

1. Center for Personalized Cancer Therapy and Division of Hematology and Oncology, Department of Medicine, University of California San Diego, Moores Cancer Center, La Jolla, CA, USA.
2. MCW Cancer Center and Genomic Sciences and Precision Medicine Center, Medical College of Wisconsin, Milwaukee, WI, USA.
3. WIN consortium, Paris, France
4. Department of Oncology, The Sidney Kimmel Comprehensive Cancer Center, The Johns Hopkins Hospital, Baltimore, MD, USA
5. Division of Hematology and Medical Oncology, Department of Internal Medicine, Seoul National University Boramae Medical Center, Seoul, Republic of Korea
6. Department of Internal Medicine, Veterans Health Service Medical Center, Seoul, Republic of Korea
7. Center for Personalized Cancer Therapy and Division of Gynecologic Oncology, Department of Obstetrics, Gynecology, and Reproductive Sciences, University of California San Diego, Moores Cancer Center, La Jolla, CA, USA.
8. Department of Surgery, Kyoto University Hospital, Kyoto, Japan
9. Division of Surgical Oncology, Department of Surgery, Center for Personalized Cancer Therapy, University of California San Diego, La Jolla, CA, USA.

Corresponding Authors

Daisuke Nishizaki, MD
Division of Hematology and Oncology
Center for Personalized Cancer Therapy
Moores Cancer Center
UC San Diego Health

3855 Health Sciences Drive
La Jolla, CA 92037
Email: dnishizaki@health.ucsd.edu

Shumei Kato, MD
Associate Professor of Medicine
Division of Hematology and Oncology
Center for Personalized Cancer Therapy
Moores Cancer Center
UC San Diego Health
Email: smkato@health.ucsd.edu

Razelle Kurzrock, MD
Professor of Medicine
Linda T. And John A. Mellowes Center for Genomic Sciences and Precision Medicine
Medical College of Wisconsin Cancer Center
Email: rkurzrock@mcw.edu

Supplemental Table 1 Characteristics of patients who underwent dual matched therapy with immunotherapy and a targeted therapy (N = 17)

**Patient characteristic**	**N=17**
Age, median (range) (years)	67 (20–86)
Sex, n (%)	
Female	9 (53%)
Male	8 (47%)
No. of prior lines, n (%)	
<3	12 (71%)
≥3	5 (29%)
Diagnosis, n (%)	
Gastroesophageal cancer	4 (24%)
Hematologic malignancies	4 (24%)
Bladder/Ureter	3 (18%)
Gynecologic	3 (18%)
Central nervous system	1 (5.9%)
Gastrointestinal stromal tumor	1 (5.9%)
Malignant mixed Müllerian tumor	1 (5.9%)
Compliance with recommendation of MTB, n (%)	
All recommended agents were given	5 (29%)
Only some recommended agents were given	12 (71%)

Abbreviations: MTB, molecular tumor board.

Supplemental Table 2 Patient characteristics including MSI status, TMB, PD-L1 IHC, and gene aberrations by NGS (N = 17)

ID (age/sex)	Cancer type	MSI status	TMB	PD-L1 IHC	Gene alterations	Summary of prior therapies ([best response], target status)
1 (67/F)	Gynecologic	MSI-high	12 Muts/Mb	Negative	*TP53* V126C, *SOX9* E50* N96fs*156, *PTCH1* S1203fs*52, *PIK3CA* P449T R88Q, *NFE2L2* D13G, *KRAS* Q61H, *FUBP1* splice site 637-1G>A, *DNMT3A* Q110fs*52, *ARID1A* G95fs*10 **(FoundationOne Tissue NGS)**	3 prior treatments<br>1. carboplatin + paclitaxel [PD]<br>2. doxorubicin [PD]<br>3. everolimus + anastrozole [SD]<br>Not previously targeted
2 (40/M)	Central nervous system	Stable	2 Muts/Mb	TC 0% IC 5% by SP142	*CDK4* amplification, *CDKN2A/B* loss, *FRS2* amplification, *JAK2* amplification, *KDR* amplification, *KIT* amplification, *MDM2* amplification, *MET* amplification, *PD-L1* amplification, *PD-L2* amplification, *PDGFRA* amplification, *RB1* splice site 2107-1G>A **(FoundationOne Tissue NGS)**	No prior treatment
3 (60/M)	Gastro-esophageal	Stable	8 Muts/Mb	TC 10% IC 1% by SP142	*ARID1A* R1276*, *CCND1* amplification, *CDK6* amplification, *FGF3* amplification, *FGF4* amplification, *FGF19* amplification, *MET* amplification, *TERC* amplification, *TP53* P278L **(FoundationOne Tissue NGS)**	1 prior treatment<br>1. FOLFOX [PD]<br>Not previously targeted
4 (84/F)	Peripheral T cell lymphoma	-	6 Muts/Mb	TC 1% IC 0% by SP142	*CREBBP* R768*, *EP300* R1529*, *NRAS* Q61R, *PTEN* Q17*, *SETD2* loss exons 19-21 **(FoundationOne Tissue NGS)**	1 prior treatment<br>1. CHOP [PD]<br>Not previously targeted
5 (54/M)	Gastro-esophageal	Stable	7 Muts/Mb	TC 1% IC 1% by SP142	*CCND1* amplification, *CDK6* amplification, *CDKN2A/B* loss, *FGF3* amplification, *FGF4* amplification, *FGF19* amplification, *PIK3CA* amplification, *PIK3CB* amplification, *PRKCI* amplification, *SOX2* amplification, *TERC* amplification, *TP53* G245D **(FoundationOne Tissue NGS)**	No prior treatment
6 (68/F)	Gynecologic	-	-	TC 0% IC 5% by SP142	*CDKN2A* G150fs*43, *NF1* R440*, *TP53* T231fs*9 **(FoundationOne cell-free DNA)**	No prior treatment
7 (86/M)	Bladder/Ureter	Stable	7 Muts/Mb	TC 0% IC 5% by SP142	*ATM* Q1627*, *CREBBP* S575fs*16, *ERBB2* amplification, *RB1* E315*, *TERT* promoter -124C>T **(FoundationOne Tissue NGS)**	1 prior treatment<br>1. Atezolizumab [SD]<br>Previously targeted
8 (72/F)	Gastrointestinal stromal tumor	-	7 Muts/Mb	Not reported	*ARID1A* truncation exon 18; *KIT* K558_E562del, N822K, V654A; *NOTCH2* P6fs*27 **(FoundationOne Tissue NGS)**	3 prior treatments:<br>1. Imatinib [unknown]<br>2. Sunitinib [CR]<br>3. Regorafenib [PD]<br>Previously targeted
9 (80/M)	Bladder/Ureter	Stable	18 Muts/Mb	Negative by SP142	*ALK* amplification, *CCNE1* amplification, *CDKN2A/B* loss, *ERBB2* amplification, *ERBB3* amplification, *KDM6A* Q240*, *MCL1* amplification, *TERT* promoter -124C>T, *TP53* Q192* **(FoundationOne Tissue NGS)**	1 prior treatment;<br>1. Carboplatin + gemcitabine [SD]<br>Not previously targeted

10 (51/F)	Gastro-esophageal	Stable	-	CPS ≥50 by 22C3	*ARID1A* Q1519fs*13, *FBXW7* splice site 502-1_507delGATGAAA, *IDH1* R132C, *MYD88* S219C, *PARK2* T240M, *RNF43* truncation intron 5, *TP53* splice site 919+1G>C **(FoundationOne Tissue NGS)**, *RHOA* Y42C **(Guardant cell-free DNA)**	3 prior treatments<br>1. FOLFOX [SD]<br>2. FOLFIRINOX [SD]<br>3. FOLFIRI [SD]<br>Not previously targeted
11 (67/M)	Gastro-esophageal	Stable	5 Muts/Mb	TC 5% IC 0% by SP142	*CCND3* amplification, *CTCF* rearrangement exon 11, *MYC* amplification, *MYST3* amplification, *RICTOR* amplification, *TP53* R175H, *TSC2* splice site 2967-2A>T, *VEGFA* amplification **(FoundationOne Tissue NGS)**	No prior treatment
12 (20/F)	Cutaneous T cell lymphoma	-	7 Muts/Mb	TC >25%, IC >25% by SP142	*CDKN2A/B* loss, *FAS* G238*, *TNFAIP3* K287fs*8/V273fs*5 **(FoundationOne Tissue NGS)**	4 prior treatments<br>1. CHOEP [unknown]<br>2. IVAC [unknown]<br>3. ICE [unknown]<br>4. Fludarabine + radiation [unknown]<br>Not previously targeted
13 (69/F)	Bladder/Ureter	Stable	14 Muts/Mb	Negative by SP142	*ERBB2* S310F, *ERBB3* E150K, *HRAS* Q61L, *STAG2* R1207fs*6, *TERT* promoter -124C>T **(FoundationOne Tissue NGS)**	3 prior treatments<br>1. Platinum [unknown]<br>2. Atezolizumab [unknown]<br>3. Carboplatin + gemcitabine [SD]<br>Previously targeted
14 (61/F)	Gynecologic	Stable	8 Muts/Mb	TC 0%, IC 5% by SP142	*CDKN2A* p16INK4a deletion and p14ARF deletion exons 2-3, p16INK4a R58*, p14ARF P72L; *FAT1* S3005*; *PIK3CA* E545K; *TERT* promoter -124C>T; *TP53* E286K **(FoundationOne Tissue NGS)**	1 prior treatment<br>1. Cisplatin + radiation [PR]<br>Not previously targeted
15 (69/F)	Malignant mixed Müllerian tumor	MSI-high	36 Muts/Mb	Negative by SP142	*ARID1A* P2005fs*10; *ARID1B* splice site 2513-2A>G; *ATM* K2337fs*20; *BRCA2* T3033fs*29; *CCND1* V293G; *CTCF* T204fs*26; *CTNNA1* R546*; *ERBB3* N126K; *EZH2* E225fs*1; *FGFR3* V677I; *FUBP1* S401fs*2; *JAK1* P430fs*2; *KRAS* G12A; *PIK3CA* G106R, R93W; *PTEN* R130P; *RNF43* G659fs*41; *SMAD4* S32fs*1 **(FoundationOne Tissue NGS)**: *MET* Q232* **(Guardant cell-free DNA)**	2 prior treatments<br>1. Carboplatin + Paclitaxel [unknown]<br>2. Ipilimumab + nivolumab [PD]<br>Previously targeted
16 (72/M)	Diffuse large B cell lymphoma	Stable	14 Muts/Mb	TPS 60% by 22C3 assay	*BCL2* G47D, *CREBBP* K663*, *DDX3X* R311fs*10, *FOXO1* T24I, *IGH* IGH-BCL2 rearrangement, *RB1* loss exons 1-12, *TP53* R273C **(FoundationOne Tissue NGS)**	1 prior treatment<br>1. RCHOP [unknown]<br>Not previously targeted
17 (37/M)	B cell lymphoma, unclassifiable	-	16 Muts/Mb	TC 50%, IC 10% by SP142	*ARID1A* G149fs*77, *FGF10* amplification, *JAK2* amplification, *KDM4C* amplification, *PD-L1* amplification, *PD-L2* amplification, *RICTOR* amplification, *SOCS1* S116N, *TNFAIP3* loss exon 3-4, *TP53* G266R **(FoundationOne Tissue NGS)**	1 prior treatment<br>1. EPOCH [unknown]<br>Not previously targeted

Abbreviations: CPS, combined positive score; CR, complete response (for solid tumors) / complete remission (for hematological malignancies): IC, immune cell; IHC, immunohistochemistry; Mb, megabase; MSI, microsatellite instability; Muts, mutations; NGS, next-generation sequencing; PR, partial response; SD, stable disease; TC, tumor cell; TPS, tumor proportion score. **Regimens**: CHOEP, cyclophosphamide + doxorubicin + etoposide + vincristine + prednisone;

CHOP, cyclophosphamide + doxorubicin + vincristine + prednisone; EPOCH, etoposide + prednisone, vincristine, cyclophosphamide, doxorubicin; FOLFIRI, folic acid + fluorouracil + irinotecan; FOLFOX, folic acid + fluorouracil + oxaliplatin; FOLFOXIRI, folic acid + fluorouracil + oxaliplatin + irinotecan; ICE, ifosfamide + carboplatin + etoposide; IVAC, ifosfamide + etoposide + cytarabine; RCHOP, rituximab + cyclophosphamide + doxorubicin + etoposide + vincristine + prednisone.

Supplemental Table 3. Administered dose and FDA-approved dose of each agent.

	Drug	Number of patients	FDA-approved dose	Administered dose	Median of dose percentage (administered dose/ FDA-approved dose)
**Immune checkpoint inhibitor**	Nivolumab	10	240mg or 3mg/kg every 2 weeks	240mg or 3mg/kg every 2 weeks (N=10)	100%
	Pembrolizumab	6	200mg every 3 weeks	200mg every 3 weeks (N=6)	100%
	Atezolizumab	1	1200 mg every 3 weeks	1200 mg every 3 weeks (N=1)	100%
**Targeted agents**	Palbociclib	4	125mg once daily 3 weeks on 1 week off	75 mg once daily 3 weeks on 1 week off (N=4)	60%
	Lenvatinib	3	24 mg once daily	10 mg once daily (N=3)	42%
	Trametinib	3	2 mg once daily	1 mg once daily (N=2), 0.5 mg once daily(N=1)	50%
	Trastuzumab	3	6 mg/kg (maintenance dose) every 3 weeks	3 mg/kg every 3 weeks (N=3)	50%
	Brentuximab	2	1.8 mg/kg every 3 weeks	1.8 mg/kg every 3 weeks (N=2)	100%
	Vismodegib	1	150 mg once daily	150 mg once daily (N=1)	100%
	Cabozantinib	1	140mg once daily	80 mg once daily (N=1)	57%
	Ibrutinib	1	420 mg daily	140 mg once daily (N=1)	33%
	Bevacizumab	1	15 mg/kg every 3 weeks	5 mg/kg every 2 weeks (N=1)	50%
	Everolimus	1	10 mg once daily	2.5 mg once daily (N=1)	25%
	Olaparib	1	300 mg twice daily	100 mg twice daily (N=1)	33%
	Venetoclax	1	Starts with 100 mg daily with dose escalation	Started with 100 mg daily with dose escalation (N=1)	100%

Abbreviations: FDA, the Food and Drugs Administration.

Supplemental Table 4. Details of three trials combining an immune checkpoint inhibitor and a gene -targeted agent and assessing biomarkers for both agents for patient inclusion

	NCT number	Country	ICI agent	Targeted agent	Cancer type	Targeted gene	Phase	Comment and citation
1	NCT 04740918	International	Atezolizumab (anti-PD-L1 inhibitor)	Trastuzumab emtansine (anti-HER2)	Breast cancer	*HER2*	3	Sponsored by Hoffmann-La Roche. "HER2+ and PD-L1+ locally advanced or metastatic breast cancer"
2	NCT 05609578	United states	Pembrolizumab	Adagrasib (KRAS G12C inhibitor)	Non-small cell lung cancer	*KRAS* G12C	2	Sponsored by Mirati. "*KRAS* G12C mutation and histologically confirmed PD-L1 TPS ≥1%"
3	NCT 05429684	China	Sintilimab (anti-PD-1 inhibitor)	Trastuzumab (anti-HER2)	Breast cancer	*HER2*	3	"Signatured by high TMB or PD-L1 positively expressed*."
4	NCT 05217446	International	Pembrolizumab	Encorafenib (BRAF inhibitor), cetuximab (EGFR inhibitor)	Metastatic colorectal cancer	*BRAF* V600E	2	"Locally confirmed microsatellite instability-high/ deficient mismatch repair (MSI-high/dMMR)." "Confirmed *BRAF* V600E mutation in tumor tissue or blood." Tumors that is RAS mutant or for which RAS mutation status is unknown are excluded.

* This study has six arms, one of which combines immune checkpoint inhibitor and targeted agent and assesses tumor mutational burden or PD-L1 positivity and HER2 IHC 3+ simultaneously for patient inclusion.

Abbreviations: IHC, immunohistochemistry; NCT, national clinical trial; PD-1, programmed death 1; PD-L1, programmed death ligand 1; TMB, tumor mutational burden; TPS, tumor proportion score.

Supplemental Figure 1 Patient flow diagram for the current study.

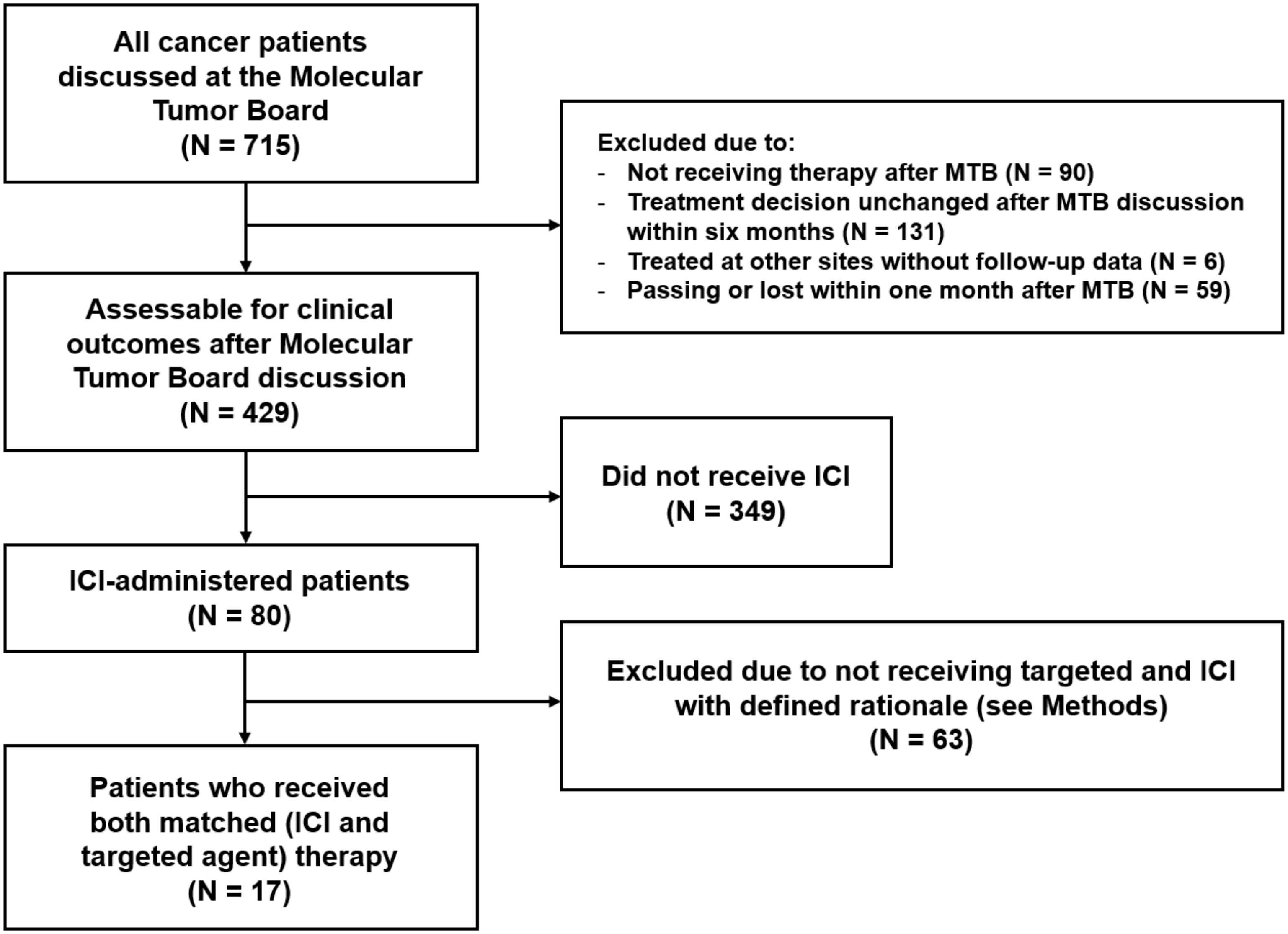

Abbreviations: ICI, immune checkpoint inhibitor; MTB, molecular tumor board.
